# (1*S*,3*S*,8*R*,9*S*,11*R*)-10,10-Di­chloro-3,7,7,11-tetra­methyl­tetra­cyclo[6.5.0.0^1,3^.0^9,11^]trideca­ne

**DOI:** 10.1107/S1600536813013457

**Published:** 2013-05-22

**Authors:** Ahmed Benharref, Najia Ourhriss, Lahcen El Ammari, Mohamed Saadi, Moha Berraho

**Affiliations:** a Laboratoire de Chimie des Substances Naturelles, ‘Unité Associé au CNRST (URAC16)’, Faculté des Sciences Semlalia, BP 2390 Bd My Abdellah, 40000 Marrakech, Morocco; b Laboratoire de Chimie du Solide, Appliquée, Faculté des Sciences, Université MohammedV-Agdal , Avenue Ibn Battouta, BP 1014, Rabat, Morocco

## Abstract

The title compound, C_17_H_26_Cl_2_, was synthesized from β-himachalene (3,5,5,9-tetra­methyl-2,4a,5,6,7,8-hexa­hydro-1*H*-benzo­cyclo­heptene), which was isolated from the essential oil of the Atlas cedar (*Cedrus Atlantica*). The asymmetric unit contains two independent mol­ecules with similar conformations. Each mol­ecule is built up from fused six- and seven-membered rings and two three-membered rings from the reaction of β-himachalene with di­chloro­carbene. In both mol­ecules, the six-membered ring has a half-chair conformation, whereas the seven-membered ring displays a boat conformation. The absolute configuration was established from anomalous dispersion effects.

## Related literature

For the reactivity and biological properties of β-himachalene, see: El Haib *et al.* (2011 ▶); El Jamili *et al.* (2002 ▶); Auhmani *et al.* (2002 ▶). For related structures, see: Oukhrib *et al.* (2013 ▶); Ourhriss *et al.* (2013 ▶). For puckering parameters, see: Cremer & Pople (1975 ▶).
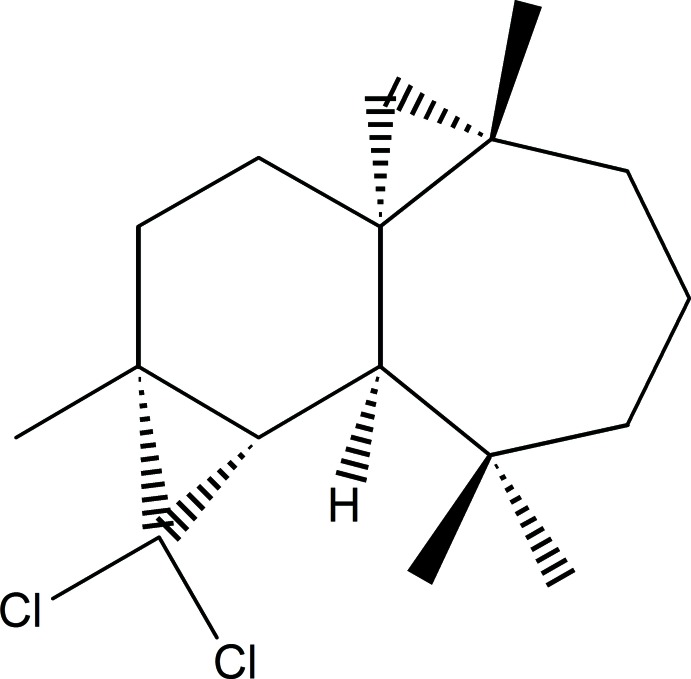


## Experimental

### 

#### Crystal data

C_17_H_26_Cl_2_
*M* *_r_* = 301.28
Monoclinic, 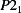

*a* = 6.4930 (2) Å
*b* = 29.0000 (8) Å
*c* = 9.2854 (4) Å
β = 110.454 (1)°
*V* = 1638.18 (10) Å^3^
*Z* = 4
Mo *K*α radiation
μ = 0.38 mm^−1^
*T* = 296 K
0.45 × 0.35 × 0.30 mm

#### Data collection

Bruker APEXII CCD diffractometer
10017 measured reflections
5832 independent reflections
5438 reflections with *I* > 2σ(*I*)
*R*_int_ = 0.021

#### Refinement

*R*[*F* ^2^ > 2σ(*F* ^2^)] = 0.050
*wR*(*F* ^2^) = 0.123
*S* = 1.09
5832 reflections
343 parameters
1 restraint
H-atom parameters constrained
Δρ_max_ = 0.29 e Å^−3^
Δρ_min_ = −0.21 e Å^−3^
Absolute structure: Flack & Bernardinelli (2000 ▶), 1283 Friedel pairs
Flack parameter: 0.05 (6)

### 

Data collection: *APEX2* (Bruker, 2009 ▶); cell refinement: *SAINT* (Bruker, 2009 ▶); data reduction: *SAINT*; program(s) used to solve structure: *SHELXS97* (Sheldrick, 2008 ▶); program(s) used to refine structure: *SHELXL97* (Sheldrick, 2008 ▶); molecular graphics: *ORTEP-3 for Windows* (Farrugia, 2012 ▶); software used to prepare material for publication: *WinGX* (Farrugia, 2012 ▶).

## Supplementary Material

Crystal structure: contains datablock(s) I, global. DOI: 10.1107/S1600536813013457/rz5066sup1.cif

Structure factors: contains datablock(s) I. DOI: 10.1107/S1600536813013457/rz5066Isup2.hkl

Supplementary material file. DOI: 10.1107/S1600536813013457/rz5066Isup3.cml

Additional supplementary materials:  crystallographic information; 3D view; checkCIF report

## Figures and Tables

**Fig. 1.**
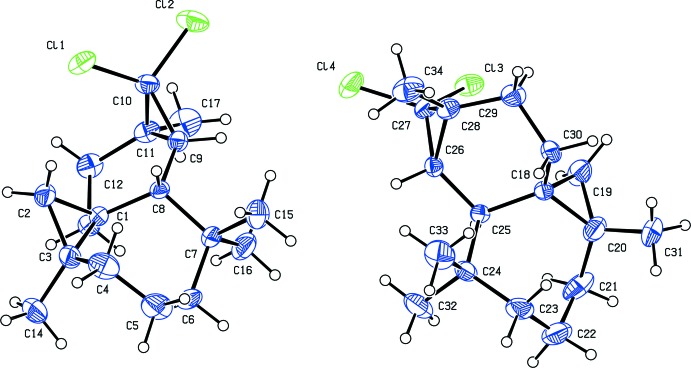
The molecular structure of the title compound with displacement ellipsoids drawn at the 30% probability level. H atoms are represented as small spheres of arbitrary radii.

## supplementary crystallographic information

### Comment

The essential oil of the Alas cedar (*Cedrus atlantica*) consist mainly (50%) of a bicyclic hydrocarbon called β-himachalene. The reactivity of this sesquiterpene and its derivatives has been studied extensively by our team (Ourhriss *et al.*, 2013; Oukhrib *et al.*,2013) in order to prepare new products having biological proprieties (El Haib *et al.*, 2011). In this work we present the crystal structure of the title compound, (1*S*,3S,8*R*,9S,11*R*)-10,10-dichloro-3,7,7,11-tetramethyltetracyclo-[6.5.0.0^1,3^.0^9,11^]tridecane.

The asymmetric unit of the title compound contains two independent molecules of similar geometry (Fig. 1). Each molecule contains a fused six- and a seven-membered rings, which is fused to two three-membered rings. The six-membered ring has a half chair conformation, as indicated by the total puckering amplitude QT = 0.492 (4) Å and spherical polar angle θ = 136.8 (5)° with φ = 131.0 (6)°, whereas the seven-membered ring displays a boat conformation with QT = 1.1447 (5) Å, θ = 88.47 (2)°, φ2 = -49.96 (2)° and φ3 = -147.50 (9)° (Cremer & Pople, 1975). Owing to the presence of Cl atoms, the absolute configuration could be fully confirmed by refining the Flack parameter (Flack & Bernardinelli, 2000) as C1(*S*), C3(*R*), C8(*R*), C9(*S*) and C11(*R*).

### Experimental

In a three-necked flask equipped with a dropping funnel, a condenser and a magnetic stirrer, maintained at 0°C, 2 g of ( 1*S*,3*R*,8*R*)-2,2-dichloro-3,7,7,10 tetramethyltricyclo[6.4.0.0^1^,^3^]dodec-9-ene (El Jamili *et al.*, 2002) were introduced in 50 ml of ether. Thereafter and simultaneously 7 g of sodium were added by small portions during one hour, and dropwise 65 ml of a methanol solution 2.5% of water. The reaction mixture was stirred for 12 h. After hydrolysis with 20 ml of water, the two phases were separated and the aqueous phase extracted three times with 20 ml of ether. The organic phases were combined and dried over sodium sulfate and concentrated. The residue obtained was chromatographed on a silica column with hexane as eluent to give the sesquiterpene hydrocarbon (1*S*,3*S*,8*R*,9*S*,11*R*)-3,7,7,11-tetramethyltricyclo-[6.5.0.^0^1,3]tridec-9-ene) with a yield of 90%. The treatment of this sesquiterpene with two equivalents of *N*-bromosuccinimide (NBS) (Auhmani *et al.*, 2002) gave the title compound with a yield of 80%. Crystals of the title compound suitable for X-ray analysis were recrystallized from pentane.

### Refinement

All H atoms were fixed geometrically and treated as riding, with C—H = 0.96-0.98 Å and with *U*_iso_(H) = 1.2*U*_eq_(C) or *U*_iso_(H) = 1.5*U*_eq_(C) for methyl H atoms.

### Crystal data

**Table:**

C_17_H_26_Cl_2_	*F*(000) = 648
*M**_r_* = 301.28	*D*_x_ = 1.222 Mg m^−^^3^
Monoclinic, *P*2_1_	Mo *K*α radiation, λ = 0.71073 Å
Hall symbol: P 2yb	Cell parameters from 5832 reflections
*a* = 6.4930 (2) Å	θ = 2.8–27.1°
*b* = 29.0000 (8) Å	µ = 0.38 mm^−^^1^
*c* = 9.2854 (4) Å	*T* = 296 K
β = 110.454 (1)°	Block, colourless
*V* = 1638.18 (10) Å^3^	0.45 × 0.35 × 0.30 mm
*Z* = 4	

### Data collection

**Table:**

Bruker APEXII CCD diffractometer	5438 reflections with *I* > 2σ(*I*)
Radiation source: fine-focus sealed tube	*R*_int_ = 0.021
Graphite monochromator	θ_max_ = 27.1°, θ_min_ = 2.8°
ω and φ scans	*h* = −8→8
10017 measured reflections	*k* = −31→37
5832 independent reflections	*l* = −11→11

### Refinement

**Table:**

Refinement on *F*^2^	Secondary atom site location: difference Fourier map
Least-squares matrix: full	Hydrogen site location: inferred from neighbouring sites
*R*[*F*^2^ > 2σ(*F*^2^)] = 0.050	H-atom parameters constrained
*wR*(*F*^2^) = 0.123	*w* = 1/[σ^2^(*F*_o_^2^) + (0.0511*P*)^2^ + 0.9439*P*] where *P* = (*F*_o_^2^ + 2*F*_c_^2^)/3
*S* = 1.09	(Δ/σ)_max_ < 0.001
5832 reflections	Δρ_max_ = 0.29 e Å^−^^3^
343 parameters	Δρ_min_ = −0.21 e Å^−^^3^
1 restraint	Absolute structure: Flack & Bernardinelli (2000), 1283 Friedel pairs
Primary atom site location: structure-invariant direct methods	Flack parameter: 0.05 (6)

### Special details

**Table:**

Geometry. All s.u.'s (except the s.u. in the dihedral angle between two l.s. planes) are estimated using the full covariance matrix. The cell s.u.'s are taken into account individually in the estimation of s.u.'s in distances, angles and torsion angles; correlations between s.u.'s in cell parameters are only used when they are defined by crystal symmetry. An approximate (isotropic) treatment of cell s.u.'s is used for estimating s.u.'s involving l.s. planes.
Refinement. Refinement of *F*^2^ against all reflections. The weighted *R*-factor *wR* and goodness of fit *S* are based on *F*^2^, conventional *R*-factors *R* are based on *F*, with *F* set to zero for negative *F*^2^. The threshold expression of *F*^2^ > 2σ(*F*^2^) is used only for calculating *R*-factors(gt) *etc*. and is not relevant to the choice of reflections for refinement. *R*-factors based on *F*^2^ are statistically about twice as large as those based on *F*, and *R*- factors based on all data will be even larger.

### Fractional atomic coordinates and isotropic or equivalent isotropic displacement parameters (Å^2^)

**Table:**

	*x*	*y*	*z*	*U*_iso_*/*U*_eq_	
C1	0.1016 (5)	0.68419 (11)	0.5418 (3)	0.0366 (7)	
C2	0.2266 (7)	0.63984 (13)	0.6031 (4)	0.0515 (9)	
H2A	0.3859	0.6411	0.6410	0.062*	
H2B	0.1642	0.6186	0.6574	0.062*	
C3	0.1069 (7)	0.64603 (13)	0.4316 (4)	0.0509 (9)	
C4	0.2490 (8)	0.65463 (17)	0.3354 (5)	0.0680 (12)	
H4A	0.3903	0.6665	0.4011	0.082*	
H4B	0.2744	0.6256	0.2926	0.082*	
C5	0.1471 (9)	0.68839 (19)	0.2059 (5)	0.0743 (14)	
H5A	0.0479	0.6718	0.1184	0.089*	
H5B	0.2624	0.7016	0.1750	0.089*	
C6	0.0183 (7)	0.72809 (16)	0.2487 (4)	0.0630 (12)	
H6A	−0.0360	0.7485	0.1608	0.076*	
H6B	−0.1089	0.7147	0.2648	0.076*	
C7	0.1415 (6)	0.75762 (13)	0.3903 (4)	0.0450 (8)	
C8	0.2305 (4)	0.72854 (10)	0.5441 (3)	0.0328 (6)	
H8	0.3810	0.7193	0.5568	0.039*	
C9	0.2458 (5)	0.75976 (11)	0.6788 (4)	0.0378 (7)	
H9	0.2630	0.7922	0.6560	0.045*	
C10	0.3447 (5)	0.74965 (13)	0.8464 (4)	0.0444 (8)	
C11	0.0997 (5)	0.75392 (13)	0.7759 (4)	0.0453 (8)	
C12	−0.0366 (6)	0.71010 (14)	0.7494 (4)	0.0513 (9)	
H12A	−0.1687	0.7160	0.7726	0.062*	
H12B	0.0468	0.6864	0.8194	0.062*	
C13	−0.1015 (5)	0.69226 (13)	0.5844 (4)	0.0482 (8)	
H13A	−0.1825	0.6636	0.5745	0.058*	
H13B	−0.1966	0.7145	0.5143	0.058*	
C14	−0.0967 (9)	0.61736 (16)	0.3506 (5)	0.0789 (15)	
H14A	−0.1805	0.6136	0.4170	0.118*	
H14B	−0.0534	0.5876	0.3256	0.118*	
H14C	−0.1849	0.6327	0.2579	0.118*	
C15	0.3343 (8)	0.78312 (17)	0.3693 (5)	0.0680 (12)	
H15A	0.4074	0.8013	0.4591	0.102*	
H15B	0.2811	0.8030	0.2812	0.102*	
H15C	0.4357	0.7612	0.3542	0.102*	
C16	−0.0308 (7)	0.79471 (16)	0.3940 (5)	0.0671 (12)	
H16A	−0.1548	0.7798	0.4076	0.101*	
H16B	−0.0784	0.8115	0.2990	0.101*	
H16C	0.0350	0.8156	0.4777	0.101*	
C17	−0.0109 (8)	0.79623 (18)	0.8130 (6)	0.0725 (13)	
H17A	−0.1668	0.7943	0.7599	0.109*	
H17B	0.0448	0.8235	0.7806	0.109*	
H17C	0.0193	0.7977	0.9217	0.109*	
C18	0.6936 (5)	0.51198 (11)	0.7358 (3)	0.0344 (6)	
C19	0.7763 (8)	0.55385 (14)	0.6770 (5)	0.0581 (10)	
H19A	0.8949	0.5496	0.6374	0.070*	
H19B	0.6698	0.5773	0.6244	0.070*	
C20	0.8290 (7)	0.54825 (14)	0.8469 (5)	0.0576 (10)	
C21	1.0606 (7)	0.53519 (19)	0.9420 (5)	0.0744 (14)	
H21A	1.1419	0.5629	0.9856	0.089*	
H21B	1.1301	0.5213	0.8755	0.089*	
C22	1.0754 (8)	0.5018 (2)	1.0714 (5)	0.0780 (15)	
H22A	1.0733	0.5192	1.1600	0.094*	
H22B	1.2148	0.4856	1.1005	0.094*	
C23	0.8863 (8)	0.46573 (17)	1.0287 (4)	0.0668 (12)	
H23A	0.9128	0.4453	1.1160	0.080*	
H23B	0.7505	0.4821	1.0153	0.080*	
C24	0.8494 (6)	0.43614 (13)	0.8876 (4)	0.0461 (8)	
C25	0.7986 (4)	0.46457 (11)	0.7330 (3)	0.0303 (6)	
H25	0.9394	0.4701	0.7193	0.036*	
C26	0.6606 (5)	0.43506 (11)	0.5985 (3)	0.0342 (6)	
H26	0.6844	0.4020	0.6202	0.041*	
C27	0.5955 (6)	0.44536 (12)	0.4307 (4)	0.0420 (7)	
C28	0.4215 (5)	0.44596 (12)	0.5037 (4)	0.0397 (7)	
C29	0.3360 (5)	0.49256 (14)	0.5310 (4)	0.0480 (8)	
H29A	0.1789	0.4904	0.5092	0.058*	
H29B	0.3598	0.5149	0.4605	0.058*	
C30	0.4486 (5)	0.50968 (12)	0.6966 (4)	0.0432 (8)	
H30A	0.3931	0.5400	0.7077	0.052*	
H30B	0.4147	0.4889	0.7672	0.052*	
C31	0.7208 (9)	0.58031 (16)	0.9285 (6)	0.0798 (15)	
H31A	0.7704	0.5728	1.0359	0.120*	
H31B	0.5641	0.5768	0.8851	0.120*	
H31C	0.7594	0.6116	0.9160	0.120*	
C32	1.0426 (8)	0.40470 (18)	0.9033 (5)	0.0735 (14)	
H32A	1.0118	0.3867	0.8113	0.110*	
H32B	1.0663	0.3845	0.9895	0.110*	
H32C	1.1719	0.4230	0.9189	0.110*	
C33	0.6514 (8)	0.40410 (17)	0.8819 (5)	0.0726 (13)	
H33A	0.6175	0.3839	0.7947	0.109*	
H33B	0.5253	0.4227	0.8731	0.109*	
H33C	0.6905	0.3861	0.9743	0.109*	
C34	0.2538 (7)	0.40760 (16)	0.4689 (5)	0.0673 (12)	
H34A	0.1552	0.4129	0.5238	0.101*	
H34B	0.3275	0.3787	0.5003	0.101*	
H34C	0.1721	0.4069	0.3605	0.101*	
Cl1	0.47292 (16)	0.69665 (3)	0.91612 (10)	0.0568 (3)	
Cl2	0.49526 (19)	0.79455 (4)	0.96635 (12)	0.0720 (3)	
Cl3	0.67805 (15)	0.49544 (3)	0.36075 (10)	0.0534 (2)	
Cl4	0.59779 (19)	0.39852 (4)	0.30805 (12)	0.0682 (3)	

### Atomic displacement parameters (Å^2^)

**Table:**

	*U*^11^	*U*^22^	*U*^33^	*U*^12^	*U*^13^	*U*^23^
C1	0.0416 (15)	0.0354 (16)	0.0306 (14)	−0.0034 (12)	0.0097 (13)	0.0025 (12)
C2	0.070 (2)	0.0372 (18)	0.0417 (18)	0.0024 (17)	0.0129 (17)	0.0019 (16)
C3	0.073 (2)	0.0385 (19)	0.0401 (17)	−0.0065 (16)	0.0186 (17)	−0.0079 (15)
C4	0.078 (3)	0.071 (3)	0.059 (3)	−0.002 (2)	0.030 (2)	−0.024 (2)
C5	0.095 (3)	0.096 (4)	0.043 (2)	−0.028 (3)	0.037 (2)	−0.016 (2)
C6	0.071 (2)	0.079 (3)	0.0291 (17)	−0.020 (2)	0.0051 (17)	0.0116 (19)
C7	0.0495 (18)	0.046 (2)	0.0351 (16)	−0.0086 (14)	0.0098 (14)	0.0099 (15)
C8	0.0271 (12)	0.0390 (17)	0.0299 (14)	−0.0022 (11)	0.0068 (11)	0.0042 (13)
C9	0.0392 (15)	0.0334 (17)	0.0387 (16)	0.0045 (12)	0.0110 (13)	0.0014 (13)
C10	0.0448 (16)	0.0470 (19)	0.0360 (17)	0.0013 (15)	0.0071 (14)	−0.0069 (15)
C11	0.0448 (17)	0.055 (2)	0.0373 (17)	0.0108 (15)	0.0153 (14)	−0.0027 (16)
C12	0.0442 (18)	0.069 (3)	0.0476 (19)	−0.0008 (16)	0.0243 (16)	0.0038 (18)
C13	0.0411 (16)	0.052 (2)	0.0459 (18)	−0.0127 (15)	0.0085 (14)	0.0053 (16)
C14	0.113 (4)	0.059 (3)	0.055 (2)	−0.033 (3)	0.016 (3)	−0.011 (2)
C15	0.074 (3)	0.070 (3)	0.066 (3)	−0.020 (2)	0.033 (2)	0.015 (2)
C16	0.060 (2)	0.064 (3)	0.066 (3)	0.011 (2)	0.008 (2)	0.026 (2)
C17	0.071 (3)	0.082 (3)	0.070 (3)	0.030 (2)	0.031 (2)	−0.008 (3)
C18	0.0427 (15)	0.0332 (15)	0.0276 (13)	0.0043 (12)	0.0127 (12)	0.0020 (12)
C19	0.085 (3)	0.043 (2)	0.047 (2)	−0.0151 (19)	0.023 (2)	0.0003 (17)
C20	0.075 (3)	0.047 (2)	0.0443 (19)	−0.0106 (18)	0.0136 (19)	−0.0099 (17)
C21	0.058 (2)	0.098 (4)	0.059 (3)	−0.032 (2)	0.011 (2)	−0.033 (3)
C22	0.071 (3)	0.106 (4)	0.043 (2)	0.018 (3)	0.002 (2)	−0.018 (3)
C23	0.087 (3)	0.081 (3)	0.0308 (17)	0.030 (2)	0.0176 (19)	0.0110 (19)
C24	0.0533 (19)	0.048 (2)	0.0360 (17)	0.0193 (16)	0.0143 (15)	0.0081 (15)
C25	0.0284 (13)	0.0369 (16)	0.0276 (13)	0.0041 (11)	0.0123 (11)	0.0012 (12)
C26	0.0371 (15)	0.0317 (16)	0.0357 (16)	0.0029 (12)	0.0151 (13)	−0.0006 (13)
C27	0.0438 (17)	0.0473 (19)	0.0337 (16)	−0.0016 (13)	0.0121 (14)	−0.0058 (14)
C28	0.0363 (15)	0.0465 (19)	0.0356 (17)	−0.0070 (13)	0.0116 (14)	−0.0016 (14)
C29	0.0315 (14)	0.062 (2)	0.0490 (18)	0.0073 (15)	0.0125 (14)	−0.0024 (18)
C30	0.0402 (16)	0.0425 (18)	0.0471 (18)	0.0129 (14)	0.0154 (14)	−0.0002 (15)
C31	0.113 (4)	0.060 (3)	0.059 (3)	0.011 (3)	0.020 (3)	−0.026 (2)
C32	0.083 (3)	0.077 (3)	0.061 (2)	0.042 (3)	0.026 (2)	0.024 (2)
C33	0.099 (3)	0.066 (3)	0.062 (3)	−0.003 (2)	0.040 (3)	0.023 (2)
C34	0.052 (2)	0.076 (3)	0.062 (2)	−0.029 (2)	0.0056 (19)	−0.003 (2)
Cl1	0.0599 (5)	0.0659 (6)	0.0385 (4)	0.0183 (4)	0.0095 (4)	0.0086 (4)
Cl2	0.0732 (6)	0.0774 (7)	0.0529 (6)	−0.0071 (6)	0.0064 (5)	−0.0284 (5)
Cl3	0.0543 (5)	0.0693 (6)	0.0364 (4)	−0.0085 (4)	0.0157 (4)	0.0074 (4)
Cl4	0.0801 (7)	0.0748 (7)	0.0458 (5)	0.0036 (5)	0.0170 (5)	−0.0248 (5)

### Geometric parameters (Å, º)

**Table:**

C1—C3	1.515 (5)	C18—C30	1.504 (4)
C1—C2	1.521 (5)	C18—C19	1.505 (5)
C1—C13	1.522 (5)	C18—C20	1.520 (5)
C1—C8	1.530 (4)	C18—C25	1.539 (4)
C2—C3	1.518 (5)	C19—C20	1.502 (6)
C2—H2A	0.9700	C19—H19A	0.9700
C2—H2B	0.9700	C19—H19B	0.9700
C3—C4	1.512 (6)	C20—C21	1.502 (6)
C3—C14	1.520 (6)	C20—C31	1.518 (6)
C4—C5	1.512 (7)	C21—C22	1.520 (7)
C4—H4A	0.9700	C21—H21A	0.9700
C4—H4B	0.9700	C21—H21B	0.9700
C5—C6	1.554 (7)	C22—C23	1.556 (8)
C5—H5A	0.9700	C22—H22A	0.9700
C5—H5B	0.9700	C22—H22B	0.9700
C6—C7	1.539 (5)	C23—C24	1.513 (6)
C6—H6A	0.9700	C23—H23A	0.9700
C6—H6B	0.9700	C23—H23B	0.9700
C7—C15	1.524 (5)	C24—C32	1.516 (5)
C7—C16	1.561 (6)	C24—C33	1.572 (6)
C7—C8	1.583 (4)	C24—C25	1.587 (4)
C8—C9	1.519 (4)	C25—C26	1.521 (4)
C8—H8	0.9800	C25—H25	0.9800
C9—C10	1.491 (5)	C26—C27	1.495 (5)
C9—C11	1.530 (5)	C26—C28	1.527 (4)
C9—H9	0.9800	C26—H26	0.9800
C10—C11	1.498 (4)	C27—C28	1.507 (5)
C10—Cl1	1.760 (4)	C27—Cl3	1.750 (4)
C10—Cl2	1.769 (3)	C27—Cl4	1.776 (4)
C11—C12	1.519 (5)	C28—C34	1.511 (5)
C11—C17	1.521 (5)	C28—C29	1.515 (5)
C12—C13	1.530 (5)	C29—C30	1.536 (5)
C12—H12A	0.9700	C29—H29A	0.9700
C12—H12B	0.9700	C29—H29B	0.9700
C13—H13A	0.9700	C30—H30A	0.9700
C13—H13B	0.9700	C30—H30B	0.9700
C14—H14A	0.9600	C31—H31A	0.9600
C14—H14B	0.9600	C31—H31B	0.9600
C14—H14C	0.9600	C31—H31C	0.9600
C15—H15A	0.9600	C32—H32A	0.9600
C15—H15B	0.9600	C32—H32B	0.9600
C15—H15C	0.9600	C32—H32C	0.9600
C16—H16A	0.9600	C33—H33A	0.9600
C16—H16B	0.9600	C33—H33B	0.9600
C16—H16C	0.9600	C33—H33C	0.9600
C17—H17A	0.9600	C34—H34A	0.9600
C17—H17B	0.9600	C34—H34B	0.9600
C17—H17C	0.9600	C34—H34C	0.9600
			
C3—C1—C2	60.0 (2)	C30—C18—C19	115.4 (3)
C3—C1—C13	120.9 (3)	C30—C18—C20	120.4 (3)
C2—C1—C13	115.5 (3)	C19—C18—C20	59.5 (2)
C3—C1—C8	118.8 (3)	C30—C18—C25	113.2 (3)
C2—C1—C8	119.2 (3)	C19—C18—C25	119.9 (3)
C13—C1—C8	112.7 (3)	C20—C18—C25	118.4 (3)
C3—C2—C1	59.8 (2)	C20—C19—C18	60.7 (2)
C3—C2—H2A	117.8	C20—C19—H19A	117.7
C1—C2—H2A	117.8	C18—C19—H19A	117.7
C3—C2—H2B	117.8	C20—C19—H19B	117.7
C1—C2—H2B	117.8	C18—C19—H19B	117.7
H2A—C2—H2B	114.9	H19A—C19—H19B	114.8
C4—C3—C1	116.1 (3)	C21—C20—C19	117.3 (4)
C4—C3—C2	116.4 (4)	C21—C20—C31	113.9 (4)
C1—C3—C2	60.2 (2)	C19—C20—C31	118.9 (4)
C4—C3—C14	113.7 (4)	C21—C20—C18	116.6 (3)
C1—C3—C14	120.9 (4)	C19—C20—C18	59.8 (2)
C2—C3—C14	119.5 (4)	C31—C20—C18	120.3 (4)
C5—C4—C3	112.9 (4)	C20—C21—C22	113.5 (4)
C5—C4—H4A	109.0	C20—C21—H21A	108.9
C3—C4—H4A	109.0	C22—C21—H21A	108.9
C5—C4—H4B	109.0	C20—C21—H21B	108.9
C3—C4—H4B	109.0	C22—C21—H21B	108.9
H4A—C4—H4B	107.8	H21A—C21—H21B	107.7
C4—C5—C6	113.6 (3)	C21—C22—C23	113.6 (3)
C4—C5—H5A	108.8	C21—C22—H22A	108.8
C6—C5—H5A	108.8	C23—C22—H22A	108.8
C4—C5—H5B	108.8	C21—C22—H22B	108.8
C6—C5—H5B	108.8	C23—C22—H22B	108.8
H5A—C5—H5B	107.7	H22A—C22—H22B	107.7
C7—C6—C5	117.7 (4)	C24—C23—C22	118.3 (4)
C7—C6—H6A	107.9	C24—C23—H23A	107.7
C5—C6—H6A	107.9	C22—C23—H23A	107.7
C7—C6—H6B	107.9	C24—C23—H23B	107.7
C5—C6—H6B	107.9	C22—C23—H23B	107.7
H6A—C6—H6B	107.2	H23A—C23—H23B	107.1
C15—C7—C6	111.4 (3)	C23—C24—C32	112.5 (3)
C15—C7—C16	107.2 (3)	C23—C24—C33	104.2 (4)
C6—C7—C16	103.9 (3)	C32—C24—C33	106.7 (4)
C15—C7—C8	108.5 (3)	C23—C24—C25	114.1 (3)
C6—C7—C8	113.0 (3)	C32—C24—C25	107.6 (3)
C16—C7—C8	112.7 (3)	C33—C24—C25	111.5 (3)
C9—C8—C1	113.0 (3)	C26—C25—C18	112.3 (2)
C9—C8—C7	108.9 (3)	C26—C25—C24	109.1 (3)
C1—C8—C7	114.2 (2)	C18—C25—C24	113.7 (2)
C9—C8—H8	106.8	C26—C25—H25	107.1
C1—C8—H8	106.8	C18—C25—H25	107.1
C7—C8—H8	106.8	C24—C25—H25	107.1
C10—C9—C8	128.5 (3)	C27—C26—C25	127.9 (3)
C10—C9—C11	59.5 (2)	C27—C26—C28	59.8 (2)
C8—C9—C11	122.9 (3)	C25—C26—C28	123.1 (3)
C10—C9—H9	112.0	C27—C26—H26	112.1
C8—C9—H9	112.0	C25—C26—H26	112.1
C11—C9—H9	112.0	C28—C26—H26	112.1
C9—C10—C11	61.6 (2)	C26—C27—C28	61.1 (2)
C9—C10—Cl1	122.1 (3)	C26—C27—Cl3	122.7 (2)
C11—C10—Cl1	121.8 (3)	C28—C27—Cl3	122.2 (3)
C9—C10—Cl2	117.0 (3)	C26—C27—Cl4	116.9 (3)
C11—C10—Cl2	118.9 (3)	C28—C27—Cl4	118.2 (2)
Cl1—C10—Cl2	108.94 (17)	Cl3—C27—Cl4	108.95 (19)
C10—C11—C12	118.1 (3)	C27—C28—C34	119.5 (3)
C10—C11—C17	118.8 (3)	C27—C28—C29	117.5 (3)
C12—C11—C17	114.7 (3)	C34—C28—C29	114.4 (3)
C10—C11—C9	59.0 (2)	C27—C28—C26	59.1 (2)
C12—C11—C9	116.3 (3)	C34—C28—C26	118.9 (3)
C17—C11—C9	118.9 (4)	C29—C28—C26	116.5 (3)
C11—C12—C13	112.7 (3)	C28—C29—C30	112.4 (3)
C11—C12—H12A	109.0	C28—C29—H29A	109.1
C13—C12—H12A	109.0	C30—C29—H29A	109.1
C11—C12—H12B	109.0	C28—C29—H29B	109.1
C13—C12—H12B	109.0	C30—C29—H29B	109.1
H12A—C12—H12B	107.8	H29A—C29—H29B	107.9
C1—C13—C12	110.6 (3)	C18—C30—C29	110.5 (3)
C1—C13—H13A	109.5	C18—C30—H30A	109.6
C12—C13—H13A	109.5	C29—C30—H30A	109.6
C1—C13—H13B	109.5	C18—C30—H30B	109.6
C12—C13—H13B	109.5	C29—C30—H30B	109.6
H13A—C13—H13B	108.1	H30A—C30—H30B	108.1
C3—C14—H14A	109.5	C20—C31—H31A	109.5
C3—C14—H14B	109.5	C20—C31—H31B	109.5
H14A—C14—H14B	109.5	H31A—C31—H31B	109.5
C3—C14—H14C	109.5	C20—C31—H31C	109.5
H14A—C14—H14C	109.5	H31A—C31—H31C	109.5
H14B—C14—H14C	109.5	H31B—C31—H31C	109.5
C7—C15—H15A	109.5	C24—C32—H32A	109.5
C7—C15—H15B	109.5	C24—C32—H32B	109.5
H15A—C15—H15B	109.5	H32A—C32—H32B	109.5
C7—C15—H15C	109.5	C24—C32—H32C	109.5
H15A—C15—H15C	109.5	H32A—C32—H32C	109.5
H15B—C15—H15C	109.5	H32B—C32—H32C	109.5
C7—C16—H16A	109.5	C24—C33—H33A	109.5
C7—C16—H16B	109.5	C24—C33—H33B	109.5
H16A—C16—H16B	109.5	H33A—C33—H33B	109.5
C7—C16—H16C	109.5	C24—C33—H33C	109.5
H16A—C16—H16C	109.5	H33A—C33—H33C	109.5
H16B—C16—H16C	109.5	H33B—C33—H33C	109.5
C11—C17—H17A	109.5	C28—C34—H34A	109.5
C11—C17—H17B	109.5	C28—C34—H34B	109.5
H17A—C17—H17B	109.5	H34A—C34—H34B	109.5
C11—C17—H17C	109.5	C28—C34—H34C	109.5
H17A—C17—H17C	109.5	H34A—C34—H34C	109.5
H17B—C17—H17C	109.5	H34B—C34—H34C	109.5
